# Phenotypic and Functional Properties of Helios^+^ Regulatory T Cells

**DOI:** 10.1371/journal.pone.0034547

**Published:** 2012-03-30

**Authors:** Daniel J. Zabransky, Christopher J. Nirschl, Nicholas M. Durham, Ben V. Park, Christina M. Ceccato, Tullia C. Bruno, Ada J. Tam, Derese Getnet, Charles G. Drake

**Affiliations:** 1 Department of Oncology, Johns Hopkins University, Baltimore, Maryland, United States of America; 2 James Buchanan Brady Urological Institute, Baltimore, Maryland, United States of America; McGill University Health Center, Canada

## Abstract

Helios, an Ikaros family transcription factor, is preferentially expressed at the mRNA and protein level in regulatory T cells. Helios expression previously appeared to be restricted to thymic-derived Treg. Consistent with recent data, we show here that Helios expression is inducible *in vitro* under certain conditions. To understand phenotypic and functional differences between Helios^+^ and Helios^−^ Treg, we profiled cell-surface markers of FoxP3^+^ Treg using unmanipulated splenocytes. We found that CD103 and GITR are expressed at high levels on a subset of Helios^+^ Treg and that a Helios^+^ Treg population could be significantly enriched by FACS sorting using these two markers. Quantitative real-time PCR (qPCR) analysis revealed increased TGF-β message in Helios^+^ Treg, consistent with the possibility that this population possesses enhanced regulatory potential. In tumor-bearing mice, we found that Helios^+^ Treg were relatively over-represented in the tumor-mass, and BrdU studies showed that, *in vivo*, Helios^+^ Treg proliferated more than Helios^−^ Treg. We hypothesized that Helios-enriched Treg might exert increased suppressive effects. Using *in vitro* suppression assays, we show that Treg function correlates with the absolute number of Helios^+^ cells in culture. Taken together, these data show that Helios^+^ Treg represent a functional subset with associated CD103 and GITR expression.

## Introduction

Regulatory T cells (Treg) are a CD4 subset that suppresses the function of multiple types of hematopoetic effector cells. This functionality most likely evolved to prevent the development of autoimmunity as a consequence of over-exuberant immune activation [1]–[5]. Correspondingly, Treg down-regulate immunity to certain pathogens [6], a property that appears to have been hijacked by tumors [5]–[7] in their efforts to escape immune surveillance. In general, Treg are characterized by the expression of the FoxP3 transcription factor [8], although some studies indicate that functional Treg can develop in the absence of Foxp3 [9]. One broad classification of Treg is based on the notion that some FoxP3 positive cells appear to be thymic-derived (natural Treg or nTreg), while other FoxP3 positive cells are induced peripherally (induced Treg or iTreg) [10].

Several microarray studies [11]–[13], including our own [14], showed a relative upregulation of the Ikaros family transcription factor Helios in Treg. In addition, two recent studies suggested that Helios expression might distinguish thymic-derived from induced Treg [15], [16]. However, this notion was recently challenged by a clear demonstration of Helios expression induced in transgenic CD4 T cells upon recognition of their cognate antigen in the presence of IL-2 and TGF-β [17]. These data suggest that the method of activation could determine Helios expression in iTreg, a finding so far unexplored in a non-TCR transgenic CD4^+^ T cell population.

A functional role for Helios in either natural or induced Treg remains unclear. Previous studies by our group have demonstrated that Helios binds to the FoxP3 promoter and upregulates FoxP3 expression [16]. Homozygous deletion of Helios was neonatally lethal in C57/Bl6 mice; the etiology for that early death remains unexplained. However, on a mixed background (129/Sv:B6), knocking out Helios did not appear to affect the absolute number of Treg or interfere with their function [18]. Using a targeted approach, Thorton *et al.* deleted Helios in CD4 cells by crossing CD4-Cre mice to Helios-fl/fl animals [15]. Consistent with the results from the genomic knockout studies, no defect in Helios-deficient Treg function was noted. Forced over-expression of Helios in Treg has not been well-described; indeed, we found that transduction of naïve human CD4 cells with a Helios expression construct appeared to induce apoptosis [16]. Based on these data, we sought to understand Helios function in Treg using an alternative approach. First we surveyed Helios^+^ versus Helios^−^ Treg for a set of cell surface markers that could enrich for Helios^+^ cells. Next, we used FACS sorting to enrich for a Helios^+^ population of Treg among naturally occurring FoxP3^+^ splenocytes, and quantified their phenotypic and functional characteristics.

## Materials and Methods

### Animals

BALB/cJ mice were purchased from The Jackson Laboratory (Bar Harbor, ME). FoxP3-GFP knock-in mice on C57BL/6 background were a generous gift of Dr. S Rudensky (Memorial Sloan Kettering Cancer Center, New York, NY). Mice were studied at 4–8 weeks of age. All animal studies were performed in accordance with protocols approved by the Animal Care and Use Committee of the Johns Hopkins University School of Medicine (animal protocol numbers MO10M44 and M009M100).

### In vitro Treg induction

Spleens and axillary lymph nodes were harvested from BALB/cJ or FoxP3-GFP mice and enriched for CD4^+^ cells via magnetic bead separation according to the manufacturer's protocol (Miltenyi Biotec, Auburn, CA). Naïve CD4 T cells (CD4^+^CD25^−^CD62L^hi^) were obtained by FACS sorting using a FACSAria II (BD, Franklin Lakes, NJ). Cells were skewed toward a Treg phenotype by activation with immobilized αCD3ε (clone 145-2c11) (5 µg/mL) and soluble αCD28 (clone 37.51) (1 µg/mL) in the presence of rTGF-β (2.5 ng/mL) and rIL2 (40 ng/mL) in RPMI as previously described or by CD3/CD28 T-activator beads (Invitrogen Dynal, Oslo), in the presence of rTGF-β (2.5 ng/mL) and rIL2 (40 ng/mL) in RPMI [14]. Stimulation by CD3/CD28 microbeads was performed in the absence of APCs.

### Flow cytometry and extracellular (ECS) and intracellular staining (ICS)

Fluorescent conjugated monoclonal antibodies were purchased from BD or eBioscience (San Diego, CA) with the exception of αHelios-FITC and αHelios-AF647 which were obtained from Biolegend (San Diego, CA). Gates and quadrants were set based on isotype control staining. MFI values were obtained using FlowJo software (Treestar, Ashland, OR) and are reported as relative MFI values using naïve CD4 T cells (CD4^+^ CD25^−^ FoxP3^−^ CD62L^hi^) as a comparison.

### Treg subset sorting

Spleens and axillary LNs were pooled from BALB/cJ mice and enriched for CD4^+^ T cells by negative selection using the mouse CD4^+^ T Cell Isolation Kit II (Miltenyi Biotec). CD4^+^ CD25^+^ cells were sorted based upon GITR and CD103 expression using the FACSAria II cell sorter (BD). Intracellular staining for FoxP3 and Helios was performed on the Treg populations obtained after sorting as per the manufacturer's protocol (eBioscience). After sorting, cells were analyzed for sorting purity and FoxP3 and Helios expression using an LSRII (BD) and FACSDiva software (BD).

### Quantitative real-time PCR

Total RNA was extracted using the RNeasy Micro Kit (Qiagen, Venlo, Netherlands) and cDNA was synthesized with the SMART PCR cDNA synthesis kit (Clontech, Mountain View, CA). All primers were purchased from Applied Biosystems (Carlsbad, CA); reactions were performed in duplicate in 2 independent experiments using an Applied Biosystems 7500 instrument. Relative mRNA frequencies were calculated in relation to 16 s mRNA expression as follows: 2 ^ΔΔ^C_t_ where ΔΔC_t_ = (ΔC_t calibration_−ΔC_t sample_).

### In vitro suppression assay

These assays were performed as previously described [19]. Briefly, spleens and axillary lymph nodes from BALB/cJ mice were pooled and enriched for CD4^+^ T cells by negative selection (Miltenyi Biotec). The CD4^−^ splenocyte fraction was collected and irradiated with 3000 rads to be used as accessory cells. The CD4^+^ T cell fraction was sorted for CD4^+^ CD25^−^ effector T cells, as well as CD4^+^CD25^+^GITR^+^CD103^−^ Tregs, CD4^+^ CD25^+^ GITR^low^ CD103^−^ Treg, and CD4^+^ CD25^+^ GITR^+^ CD103^+^ (Helios-enriched) Treg and bulk CD4^+^CD25^+^ Tregs. 2.5×10^4^ effector T cells were co-cultured with or without suppressors in various ratios in cytotoxic lymphocyte (CTL) media [14], along with 2.5×10^4^ accessory cells and were stimulated for three days with soluble αCD3 (1 µg/ml). On day three, cells were resuspended in media containing 1 µg/mL tridiated thymidine for 16 hours. H^3^ incorporation was quantified using a MicroBeta Plate Harvester and Reader (Perkin Elmer, Waltham, MA).

### Experimental tumors and *in vivo* BrdU labeling of Treg

BALB/cJ mice were injected subcutaneously with 2×10^6^ 4T1 tumor cells (American Type Culture Collection, Manassas VA). On days 8 and 9 post tumor injection, animals were injected IP with 2 mg of BrdU solution (BD). 24 hours after the second BrdU injection, mice were sacrificed and their spleens, axillary lymph nodes, tumor draining inguinal lymph nodes, and tumor infiltrating lymphocytes were isolated and stained for flow cytometric analysis. Extracellular staining was performed as previously described and cells were then incubated in Fix-Perm buffer (eBiosciences) for 16 hours. Cells were washed in Perm Buffer (eBiosciences) and were then DNAse (BD) treated for 1 hour at 37°C, washed in Perm Buffer and stained intracellularly for FoxP3, Helios, and BrdU.

### Statistical Analysis

Statistical analysis was performed using Prism 5 (GraphPad, La Jolla, CA). Unpaired two-tailed t-tests were conducted and considered significant at p-values≤0.05 (*), 0.01 (**) and 0.001 (***).

## Results

### Helios upregulation in *in vitro* induced Treg

**Figure 1:**
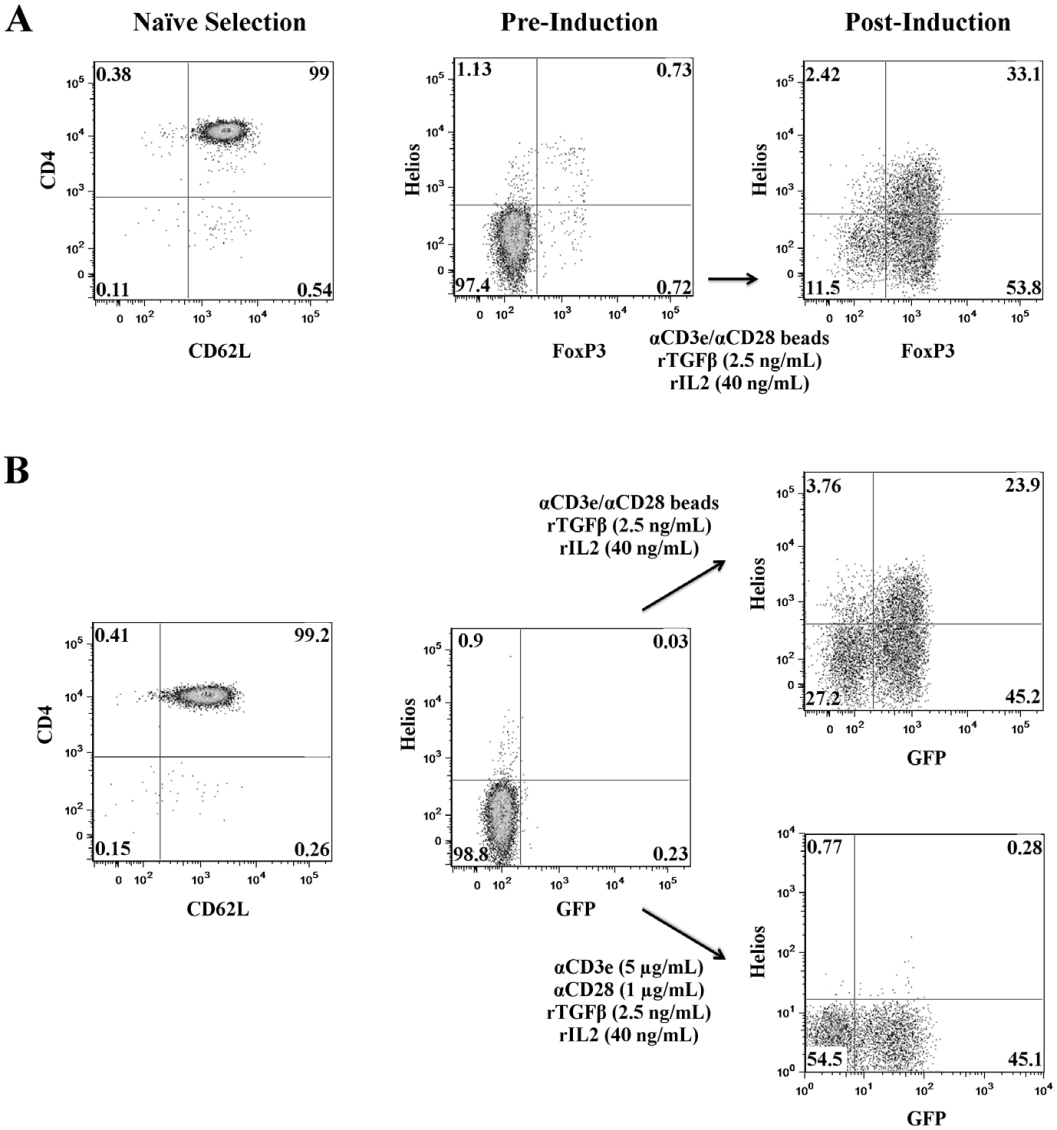
Helios up-regulation in induced Treg is determined by TCR signal. **A**) Wildtype mice: Naïve CD4^+^ T cells were purfied by sorting, and stimulated *in vitro* in the presence of TGF-β and αCD3/αCD28 microbeads. 48 hours post stimulation, Helios and FoxP3 expression was assayed by intracellular staining. Data shown are representative of 2 independent experiments, n = 5. **B**) FoxP3-GFP mice: Naïve, GFP^−^ cells were obtained by sorting and stimulated *in vitro* with either monoclonal antibodies or αCD3/αCD28 beads. As above, Helios and FoxP3 expression was assayed after 48 hours. Data shown are representative of 2 independent experiments, n = 5.

Based on recent data [17], we hypothesized that Helios expression could be induced in Treg derived from a naïve, bulk CD4 population *in vitro*. To test this hypothesis, naïve CD4 T cells were obtained from wildtype mice using CD62L as a marker for the naïve population. As shown in Figure 1A, less than 1% of these naïve T cells were positive for both FoxP3 and Helios. After 48 hours of stimulation in the presence of TGF-β and IL-2, approximately 87% of the CD4 cells expressed FoxP3. A FoxP3^+^ Helios^+^ population was clearly observed, representing 33% of total cells. Identical results were obtained using CD45RB as a marker for naïve cells (data not shown). We extended these results using FoxP3-GFP reporter mice [11] (Figure 1B), here sorting for naïve (CD62L^hi^) GFP^−^ cells. These data confirmed the observation that Helios expression indeed depends on the TCR signal provided: immobilized αCD3/soluble αCD28 induced significant FoxP3, but did not induce appreciable Helios, consistent with previous studies [15], [16]. Significantly, increasing either immobilized αCD3 or soluble αCD28 signaling was not sufficient to induce appreciable Helios expression (Figure S1). However, when TCR signaling was provided with αCD3/αCD28 microbeads in the absence of APCs, a significant percentage of cultured cells once again co-expressed both FoxP3 and Helios. Taken together these data are support a model in which Helios expression is not exclusive to natural Treg, and show that Helios expression can be induced *in vitro* under certain stimulation conditions.

### Relative over-expression of GITR and CD103 on splenic Helios+ Treg

**Figure 2:**
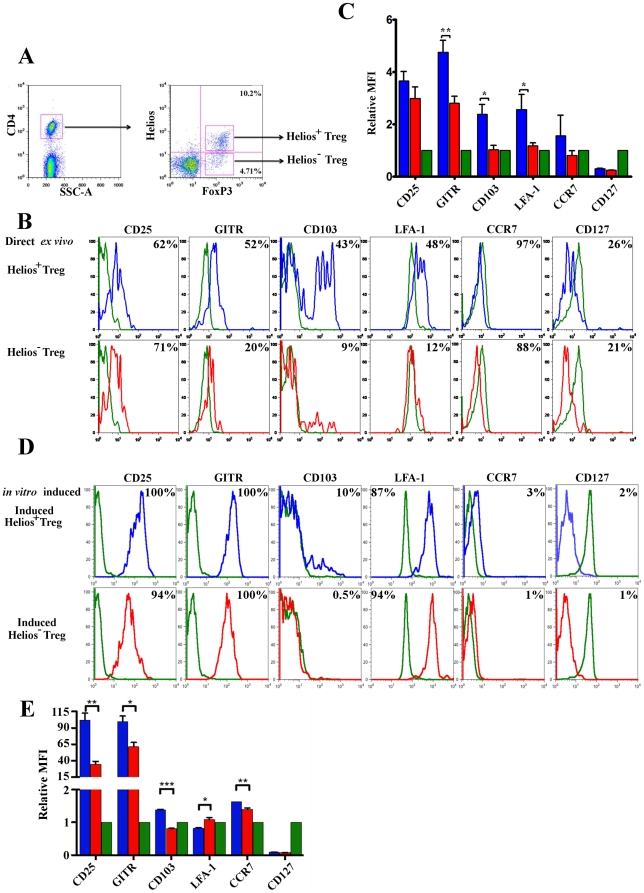
Helios^+^ and Helios^−^ FoxP3^+^ Treg cells differ in their cell surface protein expression of CD103 and GITR. **A**) Gating strategy: Unstimulated, CD4^+^ FoxP3^+^ cells were obtained from BALB/cJ splenocytes, stained and gated on the Helios^+^ and Helios^−^ populations. **B**) Direct *ex vivo* Treg: Top row: Expression of indicated cell surface molecules on CD4^+^ FoxP3^+^ Helios^+^ Treg (blue) overlaid with expression on naïve CD4 T cells (green). Bottom row: Expression on CD4^+^ FoxP3^+^ Helios^−^ Treg (red), overlaid with expression on naïve CD4^+^ cells. Percentages indicate the percentage of Treg that are positive for expression of the surface marker compared to naïve control cells. **C**) Mean Fluorescence Intensity (MFI) values for each cell surface protein in the Helios^+^ (blue) and Helios^−^ (red) Treg populations. For comparison purposes, values were normalized to the MFI of naïve, control CD4^+^ T cells (green); i.e. relative MFI values are shown. Values are +/− SEM, * (p<0.05), ** (p<0.01) comparing MFI of Helios^+^ versus Helios^−^ Treg. Data shown are representative of 3 independent experiments, n = 3/group. **D**) *In vitro* induced Treg: Top row: Expression of indicated cell surface molecules on *in vitro* induced CD4^+^ FoxP3^+^ Helios^+^ Treg (blue) overlaid with expression on naïve CD4 T cells (green). Bottom row: Expression on *in vitro* induced CD4^+^ FoxP3^+^ Helios^−^ Treg (red), overlaid with expression on naïve CD4^+^ cells. Percentages given indicate the percentage of Treg that are positive for expression of the surface marker compared to control cells. **E**) Mean Fluorescence Intensity (MFI) values for each cell surface protein in the *in vitro* induced Helios^+^ (blue) and Helios^−^ (red) Treg populations. For comparison purposes, values were normalized to the MFI of naïve, control CD4^+^ T cells (green); i.e. relative MFI values are shown. Values are +/− SEM, * (p<0.05), ** (p<0.01), *** (p<0.001) comparing MFI of Helios^+^ versus Helios^−^ *in vitro* induced Treg. Data shown are representative of 3 independent experiments, n = 3/group.

We next set out to determine the relative cell surface phenotype of Helios^+^ versus Helios^−^ Treg. CD4^+^ splenocytes obtained from wildtype BALB/c mice, and FoxP3^+^ CD4 cells were gated on the Helios^+^ versus Helios^−^ FoxP3^+^ populations (Figure 2A). We also determined the relative cell surface phenotype of *in vitro* induced Helios^+^ versus Helios^−^ Treg (Figure 2D). As expected, all four populations of Treg showed a relative increase in CD25 expression as compared to FoxP3^−^ CD4 T cells (show in in green in Figure 2B and Figure 2D). There was no significant difference in the expression of either CCR7 or CD127 between Helios^+^ and Helios^−^ Treg. Direct *ex vivo*, Helios^+^ Treg expressed LFA-1 to a significantly greater extent than Helios^−^ Treg; but the *in vitro* induced Treg showed the opposite expression pattern. Interestingly, a subset of the direct *ex vivo* Helios^+^ Treg exhibited a significantly higher level of expression of both GITR and CD103 as compared to their Helios^−^ counterparts. This trend was also observed for the *in vitro* induced Treg, but was not as pronounced. We further examined these differences in expression levels by comparing the relative MFI for each population, using the MFI of FoxP3^−^ CD4 T cells as a control (Figure 2C and 2E), finding that the MFI of GITR and CD103 was increased on Helios^+^ as opposed to Helios^−^ Treg. Taken together, these data suggest that sorting *ex vivo* CD4^+^ T cells on GITR and CD103 (in addition to CD4 and CD25) could potentially enrich a Helios^+^ FoxP3^+^ Treg population for further study. It should be noted that the relative over-expression GITR and CD103 on Helios+ versus Helios- Treg was also observed on the induced Treg as well, but those differences were small in magnitude; thus we focused our future studies on the direct *ex vivo* Treg populations.

### Helios is relatively enriched in a sorted CD103^+^ GITR^+^ Treg population

**Figure 3:**
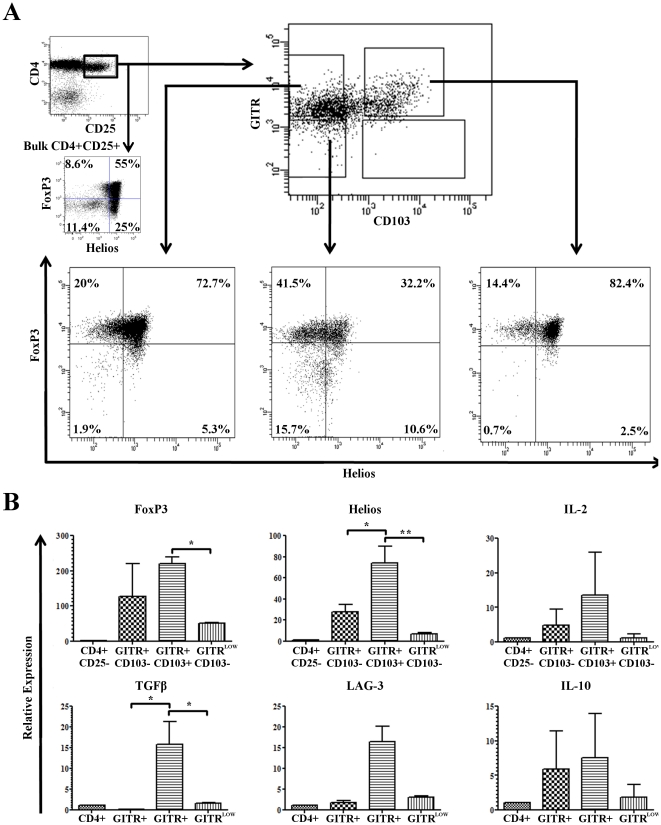
Enrichment of Helios^+^ Treg by sorting on CD103 and GITR. CD4^+^ enriched cells were FACS sorted by gating on CD4^+^ CD25^+^ cells, then sorted into three populations based on relative CD103 and GITR staining levels. Data shown are representative of 2 independent experiments, n = 5. **A**) Pre and Post sort analysis: Expression of FoxP3 and Helios is shown in both bulk CD4^+^CD25^+^ cells and sorted cells after intracellular staining. **B**) qPCR analysis: mRNA was extracted from the FACS sorted populations and reverse transcribed into cDNA. Expression of each mRNA of interest was quantified as compared to an internal control18S rRNA. Data shown are relative expression as compared to a CD4^+^ CD25^−^ reference sample. Mean values are plotted +/− SEM.

We used this difference in the expression of GITR and CD103 to enrich for Helios^+^ Treg by FACS sorting using only extracellular markers. The sorting strategy, shown in Figure 3A, was to gate on CD4^+^ CD25^+^ and then sort these cells into distinct populations based on CD103 and GITR expression. By sorting CD4^+^ CD25^+^ cells into a CD103^+^ GITR^+^ population, we were able to enrich for Helios^+^ Treg cells by approximately 2.5 fold compared to sorting for the CD4+ CD25+ CD103^−^ GITR^low^ population and by approximately 1.5 fold compared to the bulk CD4^+^CD25^+^ population. Interestingly, sorting CD4^+^ CD25^+^ cells on GITR alone provided a modest enrichment for Helios expression compared to sorting on CD4 and CD25, but not as much as by sorting on both GITR and CD103. We next explored differences in the expression of several Treg associated transcripts, at the mRNA level, in the populations obtained through sorting, comparing mRNA expression levels in the post-sort Treg populations using qPCR. For these studies, relative mRNA expression was compared to that in naïve CD4^+^ T cells (Figure 3B). The increased level of expression of Helios seen at the protein level in the CD103^+^ GITR^+^ population compared to the CD103^−^ GITR^low^ population was also observed at the mRNA level: Helios mRNA expression was tenfold higher in Helios^+^ enriched CD103^+^ GITR^+^ Treg compared to CD103^−^ GITR^low^ Treg. We found a decreased expression of FoxP3 mRNA in the CD103^−^ GITR^low^ population, which supports the finding that the CD4^+^ CD25^+^ CD103^−^ GITR^low^ population shows slightly decreased levels of FoxP3 protein expression by FACS analysis (Figure 3A). Interestingly, the Helios^+^ enriched CD103^+^ GITR^+^ population showed relatively increased expression of LAG-3, which has been suggested to be a marker of functional Treg [20], [21] at the mRNA level, but cell-surface protein levels of LAG-3 were not significantly different between Helios^+^ and Helios^−^ Treg (data not shown). Increased levels of IL-2 might reflect that Helios+ Treg represent a set of actively dividing population of Treg. In addition, we found that the CD103^+^ GITR^+^ population had a tenfold increase in TGF-β mRNA expression compared to CD103^−^ GITR^low^ Treg. This relative up-regulation of TGF-β message appeared to be associated with CD103, as opposed to GITR expression, as it was not noted in the CD103^−^ GITR^+^ population. Taken together, these data led us to hypothesize that Helios^+^ Treg might exhibit more suppressive function *in* vitro than Helios^−^ Treg.

### Proliferating Helios^+^ Treg are a major population in tumors

**Figure 4:**
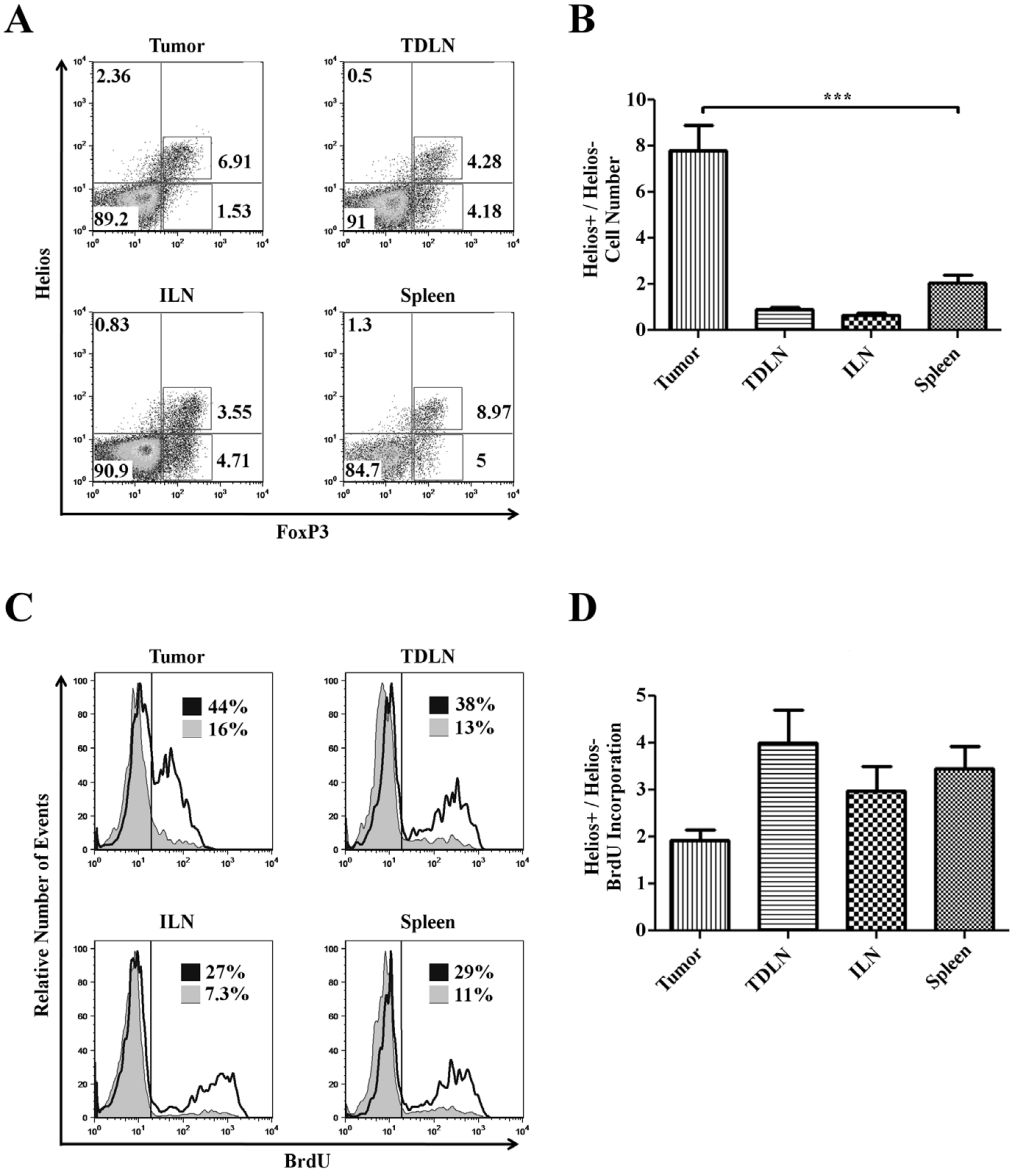
Helios+ Treg proliferate within tumors. Lymphocytes were isolated from the spleens, irrelevant lymph nodes, tumor draining lymph nodes, and tumor masses of mice bearing 4T1 tumors 10 days post implantation. Data shown are representative of 2 independent experiments, n = 5/group. **A**) Intracellular staining: lymphocytes were obtained from the indicated tissues and stained for CD4, followed by intracellular staining for Helios, FoxP3, and BrdU. **B**) Quantification of Helios^+^ versus Helios^−^ Treg. Absolute number of Helios^+^ Treg divided by absolute number of Helios^−^ Treg in given tissues, i.e. numerical ration of Helios^+^ versus Helios^−^ Treg. Data plotted are +/− SEM. **C**) Brdu incorporation into Helios^+^ (solid line) versus Helios^−^ (shaded histogram) Treg. Percentages denote the percentage of Treg from each population that are Brdu^+^. **D**) Relative proliferation of Helios^+^ Treg versus Helios^−^ Treg. MFI of BrdU staining in Helios^+^ was divided by MFI of BrdU staining in Helios^−^ Treg in given tissues. Data plotted are +/− SEM.

Given the well-documented role of Treg in attenuating an anti-tumor immune response [5], [7], we next examined the number and relative proliferation of Helios^+^ versus Helios^−^ Treg in tumor-bearing mice. To perform these studies, wildtype BALB/c mice were inoculated with 4T1 mammary tumors, and harvested 10 days after implantation. Interestingly, CD4^+^ FoxP3^+^ Helios^+^ Treg appeared to be relatively enriched in the tumor parenchyma as compared to corresponding spleens (Figure 4A). Quantitative analyses verified these observations by supporting the concept that Helios^+^ Treg are significantly more prevalent in the tumor parenchyma than are Helios^−^ Treg (Figure 4B). In non-tumor bearing mice, the ratio of Helios^+^ to Helios^−^ Treg in the spleen and axillary lymph nodes was approximately the same as in tumor-bearing mice (data not shown). More significantly, the Helios^+^ Treg in the tumor showed a greater extent of BrdU incorporation than Helios^−^ Treg in the same site (Figure 4C). This observation was not limited to the spleen; in all tissues examined Helios^+^ Treg showed greater BrdU incorporation than their Helios^−^ counterparts (Figure 4D). Interestingly, we further found that in Treg from tumors CD103 no longer distinguished between Helios^−^ and Helios^+^ Treg (data not shown), a finding consistent with recently published data [22], but which compromised our ability to perform functional analyses of Helios^+^ versus Helios^−^ Treg derived from the tumor-infiltrating population. In total, these data show that the predominant FoxP3^+^ population found within tumor parenchyma expresses Helios and proliferates more robustly in comparison to their Helios^−^ counterparts.

### Correlation of in vitro Treg suppressive function with Helios expression

**Figure 5:**
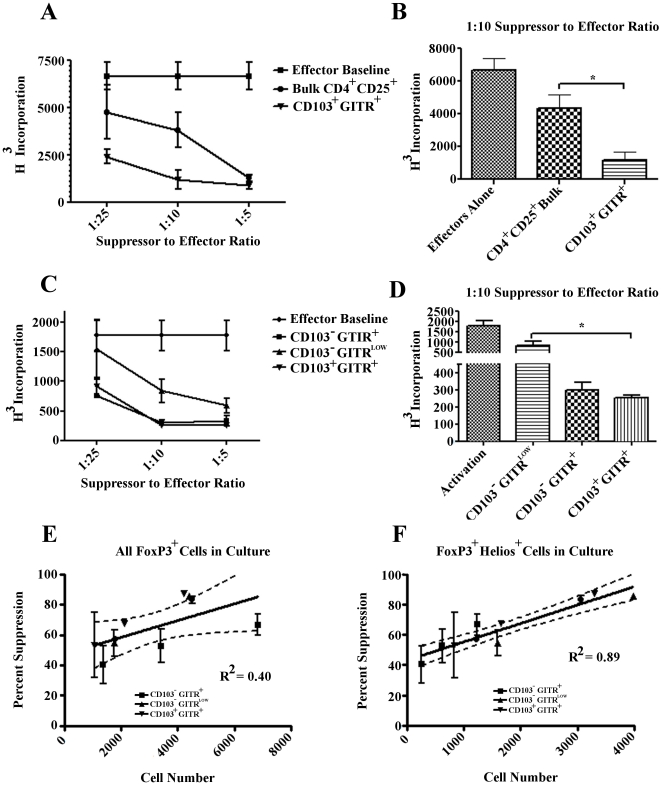
Increased suppressive function of Helios-enriched Treg populations. Standard *in vitro* suppression assays were performed using either bulk CD4^+^ CD25^+^ Treg, CD4^+^CD25^+^GITR^+^CD103^+^ Treg, CD4^+^CD25^+^GITR^low^ CD103^−^ Treg or CD4^+^CD25^+^CD103^−^GITR^+^ Treg. Data shown are representative of 2 independent experiments, n = 4. **A**) Relative proliferation of effectors by H^3^ incorporation. **B**) Analysis of Treg function at 1∶10 suppressor∶effector ratio. **C**) Relative proliferation of effectors by H^3^ incorporation. **D**) Analysis of Treg fuction at 1∶10 suppressor∶effector ratio. **E**) Percent suppression (as compared to no suppressor control) versus the absolute number of FoxP3^+^ Treg admixed. **F**) Percent suppression (as compared to no suppressor control) versus the absolute number of FoxP3^+^Helios^+^ Treg admixed.

Based on the finding that TGF-β is relatively over-expressed at the message level in Helios enriched (CD103^+^ GITR^+^) Treg, we hypothesized that these Helios-enriched Treg might demonstrate a greater level of *in vitro* suppressive capacity than a non-enriched population. To test this hypothesis, we first directly compared the suppressive capacity of Helios enriched (GITR^+^, CD103^+^) Treg to CD4+ CD25+ bulk Treg.(Figure 5A–B). As expected, at most of the suppressor to effector ratios examined, the CD4^+^CD25^+^ bulk Treg population showed a titratable suppression phenotype. However, the Helios/FoxP3 enriched CD4^+^CD25^+^CD103^+^GITR^+^ Treg population demonstrated significantly increased suppressive capabilities at most ratios examined. Furthermore, the CD4^+^CD25^+^CD103^+^GITR^+^ Tregs still showed moderate suppressive function even at a 1∶25 ratio (Figure 5A). In order to further examine the differences in the suppressive capabilities of these subpopulations, we assayed the three populations shown in Figure 3A, sorting CD4^+^CD25^+^ bulk Treg by GITR and CD103. As show in Figure 5C–D, the CD4^+^CD25^+^CD103^+^GITR^+^ Treg displayed significantly increased suppressive ability over the CD4^+^CD25^+^CD103^−^GITR^low^ population at all ratios assayed. Additionally, the CD4^+^CD25^+^CD103^+^GITR^+^ Tregs showed a small increase in suppressive ability over the CD4^+^CD25^+^CD103^−^GITR^+^ Tregs. These differences in the suppressive capacity of the sorted Treg populations could be due to increases in the number of cells in each population expressing FoxP3, Helios, or both. To examine this question, we compared the percent suppression observed to the number of FoxP3^+^ singly positive (Figure 5E) or FoxP3^+^Helios^+^ doubly positive (Figure 5F) Tregs in culture (based on post sort staining percentages). We were surprised to find very little correlation (R^2^ = 0.40) between the number of FoxP3^+^ Tregs in culture and percentage suppression (Figure 5E). However, percentage suppression correlated strongly with the number of FoxP3^+^, Helios^+^ cells (R^2^ = 0.89, Figure 5F). Taken together, these data show that several suppressor populations exist within the bulk CD4^+^CD25^+^ Treg population, and that enriching for FoxP3^+^Helios^+^ Tregs results in an increased *in vitro* suppressive capability.

## Discussion

Microarray data from several groups [11]–[13], including our own [14], showed increased expression of the Ikaros family transcription factor Helios in regulatory T cells (Treg). Recently, Thorton *et al.* [15] utilized a new monoclonal antibody to confirm these observations at the protein level. This group further suggested that Helios expression might serve to distinguish natural (thymic-derived) from peripherally derived Treg, a finding consistent with our data as well [16]. However, the notion that Helios could distinguish between those two populations was subsequently challenged by data showing Helios expression in TCR transgenic cells induced toward a Treg phenotype *in vitro*. Here we extend those more recent data, showing that *in vitro* stimulation of naïve CD4 T cells in the presence of IL-2 and TGF-β can lead to the development of both Helios^+^ and Helios^−^ FoxP3^+^ Treg.

The function, if any, of Helios in Treg remains relatively unknown. Indeed, either global Helios knockout [18] or CD4-targeted Helios knockout [15] mice showed no overt deficiency in Treg number or function. Our efforts to over-express Helios in naïve CD4 cells have been, to date, thwarted by the observation that successful expression seems to induce apoptosis [16]. We thus set out to study the Helios^+^ population of Treg by elucidating a set of cell surface markers that could enrich CD4^+^ CD25^+^ cells for a Helios-expressing population. Interestingly, we found that expression of the glucocorticoid-induced TNF receptor (GITR), a well-accepted Treg molecule [23], correlated with Helios expression in unstimulated splenic Treg. These data represented our first data suggesting that Helios^+^ Treg might represent an activated, functional population. CD103, an α/β integrin associated with gut-homing of lymphocytes [24], and preferentially expressed on tumor-infiltrating Treg [22] was also relatively up-regulated on Helios^+^ versus Helios^−^ Treg, again suggesting the potential for an increased functional capacity [25], [26]. These data are consistent with a recent large-scale microarray analysis of Treg subtypes, which also showed that Helios message correlates with CD103 expression [27].

By sorting unstimulated Treg from the spleens of unmanipulated wild-type mice on CD4, CD25, GITR and CD103, we were able to isolate a FoxP3^+^ Treg population relatively enriched for Helios expression. Our results must be tempered by the notion that this was a relative (2 to 3) fold over-expression as compared to GITR^low^ CD103^−^ Treg, and that the isolation of a pure Helios^+^ population will most likely require the generation of an appropriate reporter strain of mice. Nevertheless, qPCR studies showed a relative up-regulation of TGF-β message in Helios-enriched Treg, providing a second indication that Helios expression in Treg might correlate with suppressive function.

We next turned to a tumor model and found that Treg in the tumor-infiltrating population were dramatically enriched for Helios^+^ cells. BrdU labeling studies showed that Helios^+^ Treg were proliferating *in vivo* to a greater exent than were Helios^−^ Treg. Unfortunately, our efforts to enrich Helios^+^ Treg from the tumor bed were complicated by the finding that both Helios^+^ and Helios^−^ Treg expressed similar levels of CD103 and GITR at this location. While disappointing from an experimental standpoint, these data are in excellent agreement with recent studies showing that CD103 marks Treg in a tumor bed [22], and once again the support the possibility that Helios-enriched, CD103^+^ GITR^+^ Treg might represent a population with a relatively enhanced suppressive capacity.

Using an *in vitro* suppression assay, we confirmed this hypothesis, finding that CD103^+^ GITR^+^ CD4^+^ CD25^+^ (Helios-enriched) Treg mediated a greater degree of suppression than CD4^+^ CD25^+^ Treg, or GITR^low^ CD103^−^ Treg. It has been previously shown that CD103^+^ Treg are more potent suppressors than CD103^−^ Treg [25], [28], [23], and our data suggests that this difference may correlate with Helios expression. Interestingly, the degree of suppression in any of our Treg assays was closely proportional to the absolute number of Helios^+^ cells admixed, providing reasonable support for the notion that Helios may be a marker of Treg with functional, suppressive capacity. Our conclusions, however, must be interpreted in the light of recent data, from two separate groups, showing that Helios knockout did not affect Treg number or function [15]–[18]. One interpretation of these findings is that Helios expression in Treg is a non-essential correlate of CD103 and GITR expression, and is thus not at all necessary for Treg activity *in vivo*. Another possibility is that multiple Ikaros family members operate in concert to modulate Treg function, as has been observed for Ikaros and Helios in determining SHIP expression in B cells [29]. Despite these caveats, our data support the notion that Helios expression, while likely not absolutely required for Treg function, correlates with the functional capacity of Treg *in vitro*.

## Supporting Information

Figure S1 **Increasing concentrations of α-CD3 and/or α-CD28 do not result in significant Helios induction in vitro.** **A**) Experiments were performed in the same fashion as in Figure 1B.Cells were stimulated with either 0, 5, 10, or 25 µg/mL of plate bound α-CD3 and 1.25 µg/mL of soluble α-CD28. **B**) Cells were stimulated with either 0, 5, 10, or 25 µg/mL of soluble α-CD28 and 1.25 µg/mL of plate bound α-CD3. **C**) Cells were stimulated with 0, 5, 10, or 25 µg/mL of both plate bound α-CD3 and soluble α-CD28.
(TIF)
