# Supplementary figures and images for: Phenotypic and Functional Properties of Helios+ Regulatory T Cells
Source: PLoS One. 2012 Mar 30;7(3):e34547. doi: 10.1371/journal.pone.0034547 (PMC3316700; doi:10.1371/journal.pone.0034547)

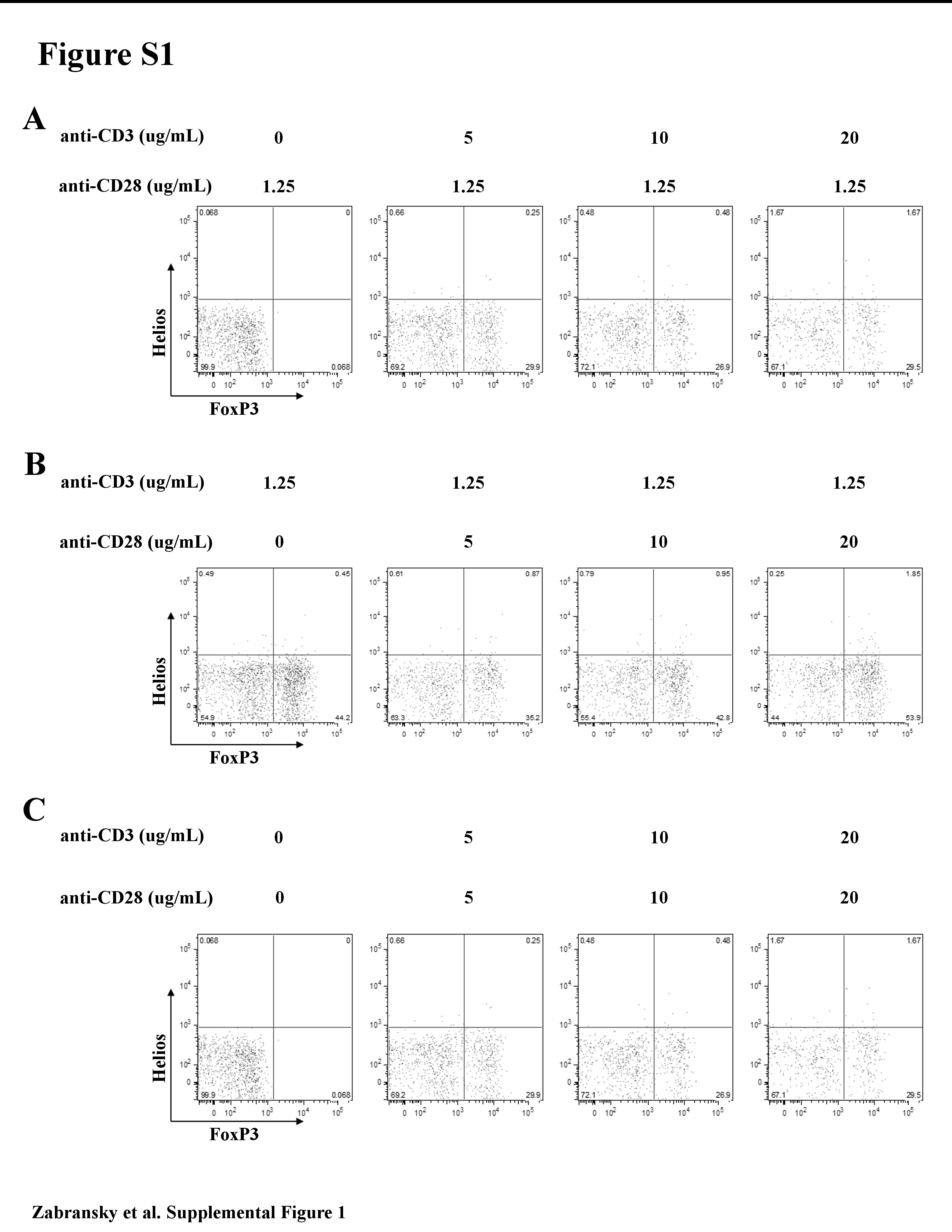

Supplement: Figure S1 — Increasing concentrations of α-CD3 and/or α-CD28 do not result in significant Helios induction in vitro. A) Experiments were performed in the same fashion as in Figure 1B.Cells were stimulated with either 0, 5, 10, or 25 µg/mL of plate bound α-CD3 and 1.25 µg/mL of soluble α-CD28. B) Cells were stimulated with either 0, 5, 10, or 25 µg/mL of soluble α-CD28 and 1.25 µg/mL of plate bound α-CD3. C) Cells were stimulated with 0, 5, 10, or 25 µg/mL of both plate bound α-CD3 and soluble α-CD28. (TIF) [file pone.0034547.s001.tif]
